# Dietary Organic Selenium Supplementation for Weaned Piglets Challenged with Deoxynivalenol

**DOI:** 10.3390/ani15172546

**Published:** 2025-08-29

**Authors:** Wenyue Zhou, Haopeng Zhong, Zhouyin Huang, Jiajun Han, Zheng Yang, Tiande Zou, Jinming You, Jun Chen

**Affiliations:** 1 Jiangxi Province Key Laboratory of Animal Nutrition and Feed, Jiangxi Province Key Innovation Center of Integration in Production and Education for High-Quality and Safe Livestock and Poultry, Jiangxi Agricultural University, Nanchang 330045, China; 2 Ganzhou Animal Husbandry and Fisheries Research Institute, Ganzhou 341000, China

**Keywords:** bioavailability, intestinal health, microbiota modulation, mycotoxin, selenomethionine, swine

## Abstract

This study aimed to investigate the effects of dietary selenium (Se) supplementation levels on the apparent total tract digestibility (ATTD) of crude protein, crude fat, and crude ash, and cecal microbiota diversity and composition in nursery piglets challenged with deoxynivalenol (DON). Twenty-four weaned piglets were allocated to four dietary treatment groups (*n* = 6). The four treatment diets were as follows: (1) post-weaning diet containing 0.3 mg/kg of Se; (2) post-weaning diet without supplemental Se + 3 mg/kg DON; (3) post-weaning diet containing 0.3 mg/kg Se + 3 mg/kg DON; and (4) post-weaning diet containing 0.5 mg/kg Se + 3 mg/kg DON. The native Se levels in the feedstuffs were 0.03–0.06 mg/kg. The Se source was supplemented in the form of selenomethionine. The trial lasted 28 days, with fecal samples collected during the final 4 days for ATTD analysis of crude protein, crude fat, and crude ash. Upon trial completion, cecal digesta was sampled for microbial diversity and composition analysis. The results showed that piglets in the 0.5 mg/kg Se + DON group had a higher ATTD of crude fat than the 0 mg/kg Se + DON group and a greater ATTD of crude ash than the 0.3 mg/kg Se + DON group (*p* < 0.05). Additionally, the 0.5 mg/kg Se + DON group displayed increased Shannon and Chao 1 indices compared to the 0 mg/kg Se + DON group (*p* < 0.05). Microbial composition analysis revealed higher Firmicutes abundance in the 0.5 mg/kg Se + DON group than in the 0.3 mg/kg Se + DON group, along with reduced Euryarchaeota abundance relative to the 0 mg/kg Se + DON group (*p* < 0.05). The 0.5 mg/kg Se + DON group also had higher Selenomonadaceae abundance than the other groups (*p* < 0.05). Pearson correlation analysis indicated a negative correlation between Selenomonadaceae abundance and the ATTD of ash, whereas [Eubacterium]_coprostanoligenes_group abundance was positively correlated with the ATTD of ash (*p* < 0.10). Collectively, dietary Se supplementation at 0.5 mg/kg improved nutrient digestibility in DON-exposed nursery piglets, which was potentially associated with the modulation of cecal microbiota diversity and composition.

## Simple Summary

The consumption of deoxynivalenol (DON) commonly induces multiple detrimental effects in swine, including diminished nutrient digestibility. Emerging evidence suggests that gut microbiota may play a critical role in mediating the toxicological impact of DON in animals. In pigs, the cecum serves as the primary site for microbiota digestibility. Consequently, it is imperative to explore potential approaches to mitigate impaired nutrient digestibility and disturbances in cecal microbiota in pigs exposed to DON-contaminated feed. Importantly, in this study, dietary selenium supplementation at 0.5 mg/kg elevated the apparent total tract digestibility of crude ash in DON-exposed nursery piglets, which may correlate with alterations in cecal microbial diversity and composition.

## 1. Introduction

In modern pig production, feed costs constitute approximately 60% to 70% of total expenses [1]. Meanwhile, feed contamination by mycotoxins is a very common issue, with deoxynivalenol (DON) being particularly prevalent [2]. DON, a type B trichothecene mycotoxin, is primarily produced by *Fusarium graminearum* and *Fusarium culmorum* [3]. A decade-long survey on mycotoxins in animal feed revealed an 84.8% detection rate of DON in 13,232 feed samples across East Asia [4]. Pigs are the most sensitive animals to DON [3]. Ingestion of DON typically results in various adverse effects on pigs, including reduced nutrient digestibility [5,6]. More importantly, the gut microbiota has been increasingly recognized as a potential mechanism underlying the toxicity of DON in animals [7,8,9]. The cecum is the main site of microbiota digestibility in pigs [10]. It has been demonstrated that exposure to DON can result in disturbances of the cecal microbiota in weaned piglets [9,11]. Therefore, it is essential to investigate potential intervention strategies for compromised nutrient digestibility and cecal microbiota disruptions in pigs fed DON-contaminated diets.

Selenium (Se) is an essential trace element for swine [12,13]. Se has been shown to mitigate the toxic effects of DON in pigs, including intestinal [14,15], hepatic [16], and splenic toxicity [17,18,19]. Interestingly, research indicates that dietary Se supplementation can modulate gut microbiota and thus enhance host functions and health in various contexts, including the intestinal health [20,21] and testicular function [22] in murine models. In the swine industry, increasing evidence suggests the potential role of gut microbiota-mediated protective effects of Se on weaned piglets [23,24], growing–finishing pigs [25], finishing pigs with ammonia challenge [26], and gilts [27]. Given the established links between Se, gut microbiota modulation, and the mitigation of DON toxicity, it is hypothesized that Se’s protective effects in nursery piglets fed DON-contaminated diets may also be mediated through the cecal microbiome. Therefore, the present study was conducted to investigate the effects of different levels of dietary Se supplementation on the apparent total tract digestibility of crude protein, crude fat, and crude ash, and cecal microbiota diversity and composition in nursery piglets challenged with DON.

## 2. Materials and Methods

### 2.1. Experimental Design

Twenty-four Duroc × Landrace × Yorkshire weaned male piglets (28-day age), with an average weight of 6.78 kg, were allocated into four dietary treatments by completely randomized design, with six replicates per treatment and one pig per replicate. The four treatment diets were as follows: (1) post-weaning diet containing 0.3 mg/kg of Se; (2) post-weaning diet without supplemental Se + 3 mg/kg DON; (3) post-weaning diet containing 0.3 mg/kg Se + 3 mg/kg DON; and (4) post-weaning diet containing 0.5 mg/kg Se + 3 mg/kg DON. Native Se levels in the feedstuffs were 0.03–0.06 mg/kg. Se was supplemented as selenomethionine (purity ≥ 99%, Sigma Aldrich, St. Louis, MO, USA). DON was included in the diets as commercially purified DON (purity ≥ 98%, Shanghai Yujing Biotechnology, Shanghai, China). The rationale for selecting a DON dosage of 3 mg/kg was based on the study by Bracarense et al. (2012) [28]. The study lasted for 28 days, during which 24 piglets were individually housed in metabolism cages (0.8 m height × 1.5 m length × 1.0 m width), each fitted with a nipple drinker and a feeder. The experimental diets were formulated to meet the nutritional requirements of piglets (NRC, 2012) [29], except for Se in the DON group without Se supplementation. The experimental diets and feeding management protocols were referenced from our previous study [16]. The dietary treatment groups are outlined in Table 1. The pigs had ad libitum access to fresh water. The piglets received experimental diets, with standardized daily feed allotments of 320, 400, 500, and 600 g over periods of 1, 2, 3, and 4 weeks, respectively, to ensure consistency and reduce variability in feed intake.

### 2.2. Sample Collection

On days 25–28, fecal samples were collected twice daily, with 10 mL of a 10% H_2_SO_4_ solution added per 100 g of sample. Following the four-day collection period, equal aliquots from each pig were homogenized into a composite sample and stored at −20 °C for subsequent determination of the apparent total tract digestibility (ATTD). Concurrently, feed samples representing each dietary treatment were collected and uniformly combined into a single pooled sample for further analysis.

At the end of the feeding trial, all pigs were anesthetized with sodium pentobarbital and subsequently slaughtered. The entire cecum was removed, and cecum digesta samples were collected using a 1 mL pipette with a 1 mL pipette tip. Approximately 500 mg of cecum digesta was collected per pig, transferred into cryogenic storage tubes, and stored at −80 °C until microbiota analysis.

### 2.3. Laboratory Analysis

#### 2.3.1. ATTD Analysis

The ATTD of crude protein, crude fat, and crude ash was determined through the acid-insoluble ash method [30,31]. The contents of crude protein, crude fat, and crude ash in both feed samples and fecal samples were determined in accordance with the analytical procedures outlined by AOAC (1995) [32]. The ATTD was calculated as follows: ATTD = {1 − [(AD × NF)/(AF × ND)]} × 100%, where AD and AF represent the acid-insoluble ash content in the diets and feces, respectively, and ND and NF represent the nutrient content in the diets and feces, respectively [31].

#### 2.3.2. Cecum Microbiota Analysis

The cecal microbiota was analyzed via 16S rRNA sequencing. Briefly, genomic DNA was isolated from the cecal digesta samples, followed by amplification of bacterial 16S rRNA genes (V3–V4 region). The resulting PCR amplicons were purified and subjected to sequencing on the Illumina HiSeq2500 platform (Illumina, San Diego, CA, USA). Subsequent data processing and bioinformatics analysis were conducted by NovoGene Bioinformatics Technology Co., Ltd. (Beijing, China). Briefly, the bioinformatics analysis was performed using the QIIME 2 platform. α-diversity metrics, comprising the Shannon, Chao 1, and Simpson indices, were calculated. β-diversity was assessed through weighted UniFrac distances and analyzed via permutational multivariate analysis of variance with the Adonis procedure. Relative abundances of the top ten bacterial phyla and fifteen bacterial families were determined using the taxa plugin. LEfSe analysis was employed to identify differentially abundant taxa, with an LDA score cutoff of 3. Microbial functional profiles were predicted at metabolic levels 2 and 3 from 16S rRNA sequences using the PICRUSt v2.6.2 software.

### 2.4. Statistical Analysis

Individual pigs were designated as the experimental units. For the ATTD of crude protein, crude fat, and crude ash, data were analyzed using one-way ANOVA followed by Duncan’s multiple range test (SPSS 25.0, Chicago, IL, USA). Microbial α-diversity and β-diversity were analyzed using the R package vegan 2.5-6, and microbial composition was analyzed with R 3.5.1. Pearson correlation analysis was performed to evaluate the relationship between cecal microbiota composition and the ATTD of the measured nutrients (SPSS 25.0, Chicago, IL, USA). Statistical significance was defined as a *p*-value below 0.05, whereas values between 0.05 and 0.10 were interpreted as indicative of a trend toward significance.

## 3. Results

### 3.1. ATTD

**Figure 1:**
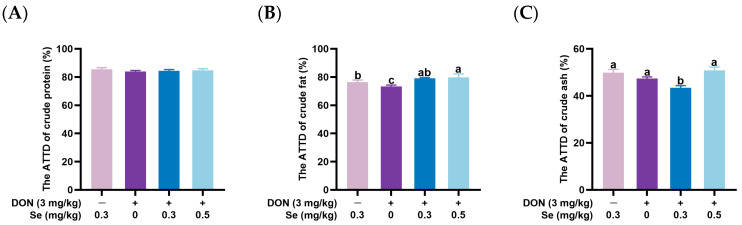
Effects of dietary selenium (Se) supplementation levels on the apparent total tract digestibility (ATTD) of nursery piglets exposed to deoxynivalenol (DON) (*n* = 6, mean ± SEM). (**A**) The ATTD of crude protein. (**B**) The ATTD of crude fat. (**C**) The ATTD of crude ash. Different letters above the bars indicate statistical differences among the treatment groups (*p* < 0.05).

As illustrated in Figure 1, the ATTD of crude protein in nursery piglets was unaffected by the dietary treatments (*p* > 0.05). However, compared with the 0.3 mg/kg Se group, piglets in the 0.3 mg/kg Se + deoxynivalenol (DON) exhibited a reduced ATTD of crude ash, while those in the 0 mg/kg Se + DON group showed a decreased ATTD of crude fat (*p* < 0.05). Additionally, piglets in the 0.5 mg/kg Se + DON group demonstrated a higher ATTD of crude fat compared to the 0 mg/kg Se + DON group and a greater ATTD of crude ash relative to the 0.3 mg/kg Se + DON group (*p* < 0.05).

### 3.2. Diversity of Cecum Microbiota

**Figure 2:**
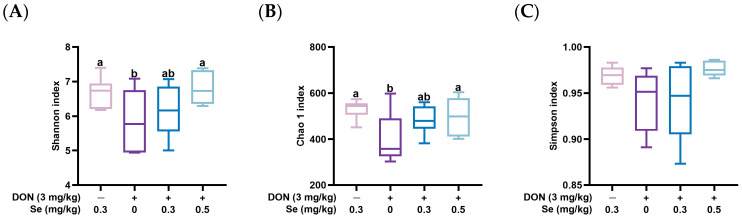
Effects of dietary selenium (Se) supplementation levels on the α-diversity of cecum microbiota of nursery piglets exposed to deoxynivalenol (DON) (*n* = 6). (**A**) Shannon index. (**B**) Chao 1 index. (**C**) Simpson index. Different letters above the bars indicate statistical differences among the treatment groups (*p* < 0.05).

**Figure 3:**
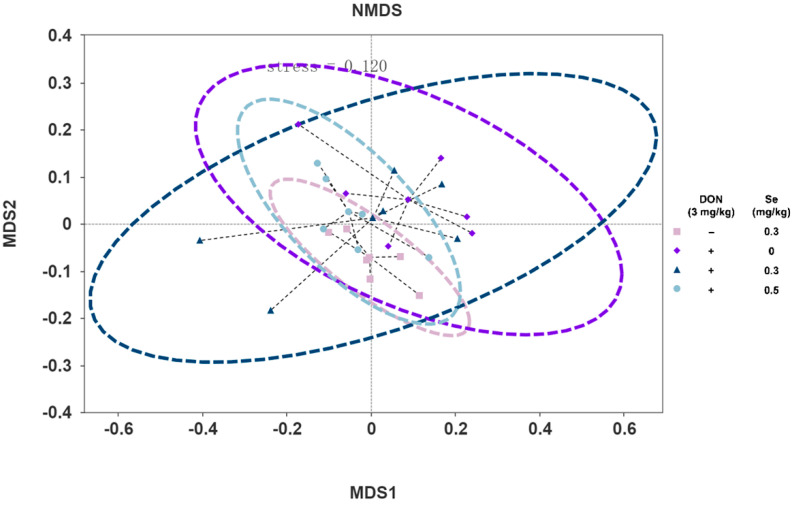
Effects of dietary selenium (Se) supplementation levels on the β-diversity of cecum microbiota of nursery piglets exposed to deoxynivalenol (DON) (*n* = 6). Statistical differences among treatment groups using Adonis analysis: 0.3 mg/kg Se + DON group vs. 0.3 mg/kg Se group: *p* = 0.020; 0.3 mg/kg Se + DON group vs. 0.5 mg/kg Se + DON group: *p* = 0.905; 0.3 mg/kg Se + DON group vs. 0 mg/kg Se + DON group: *p* = 0.308; 0.5 mg/kg Se + DON group vs. 0 mg/kg Se + DON group: *p* = 0.279.

Figure 2 displays the effects of the dietary Se supplementation levels on the α-diversity of the cecum microbiota of nursery piglets exposed to DON. The Simpson index of the cecum microbiota in nursery piglets was not influenced by the dietary treatments (*p* > 0.05). However, compared with the 0.3 mg/kg Se group, the Shannon index and Chao 1 index were decreased in the cecum microbiota of nursery piglets from the 0 mg/kg Se + DON group (*p* < 0.05). In contrast, compared to the 0 mg/kg Se + DON group, the Shannon index and Chao 1 index were increased in the cecum microbiota of nursery piglets from the 0.5 mg/kg Se + DON group (*p* < 0.05). Furthermore, a statistically significant difference was observed in the β-diversity of the cecum microbiota in piglets between the 0.3 mg/kg Se group and the 0.3 mg/kg Se + DON group (*p* < 0.05) (Figure 3).

### 3.3. Composition of Cecum Microbiota

**Figure 4:**
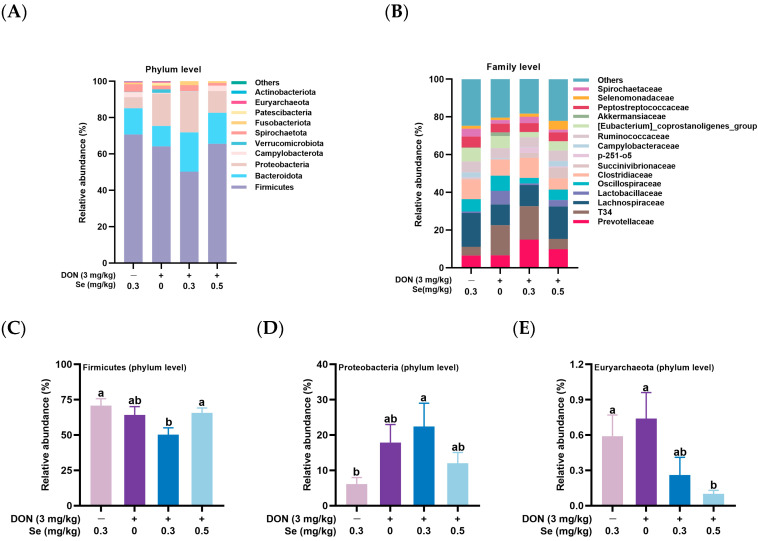

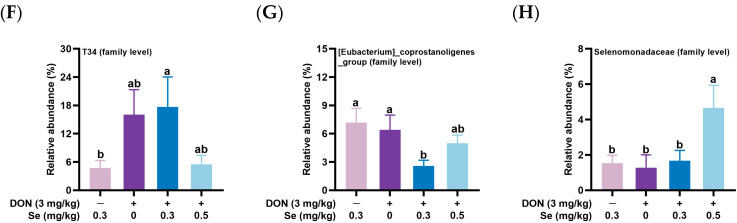
Cecum microbial composition at phylum (top 10) and family (top 15) levels of nursery piglets in response to the dietary treatments (*n* = 6). (**A**,**B**) Cecum microbial composition at phylum level (top 10) and at family level (top 15). (**C**–**H**) Relative abundance of Firmicutes, Proteobacteria, Euryarchaeota, T34, [Eubacterium]_coprostanoligenes_group, and Selenomonadaceae (mean ± SEM). Different letters above the bars indicate statistical differences among the treatment groups (*p* < 0.05).

Figure 4 illustrates the cecal microbial composition at the phylum level (top 10 phyla) and family level (top 15 families) in the nursery piglets in response to the dietary treatments. At the phylum level, compared with the 0.3 mg/kg Se group, piglets in the 0.3 mg/kg Se + DON group had a decreased relative abundance of Firmicutes and increased relative abundance of the Proteobacteria in the cecum (*p* < 0.05). Additionally, piglets in the 0.5 mg/kg Se + DON group had a higher relative abundance of Firmicutes compared to the 0.3 mg/kg Se + DON group, and a lower relative abundance of Euryarchaeota compared to the 0 mg/kg Se + DON group (*p* < 0.05). At the family level, compared with the 0.3 mg/kg Se group, piglets in the 0.3 mg/kg Se + DON group had a higher relative abundance of T34 and lower relative abundance of [Eubacterium]_coprostanoligenes_group in the cecum (*p* < 0.05). Furthermore, compared to the 0.3 mg/kg Se + DON group, piglets in the 0 mg/kg Se + DON group had an increased relative abundance of [Eubacterium]_coprostanoligenes_group in the cecum (*p* < 0.05). Additionally, the relative abundance of Selenomonadaceae was higher in piglets from the 0.5 mg/kg Se + DON group compared to the other three treatment groups (*p* < 0.05).

**Figure 5:**
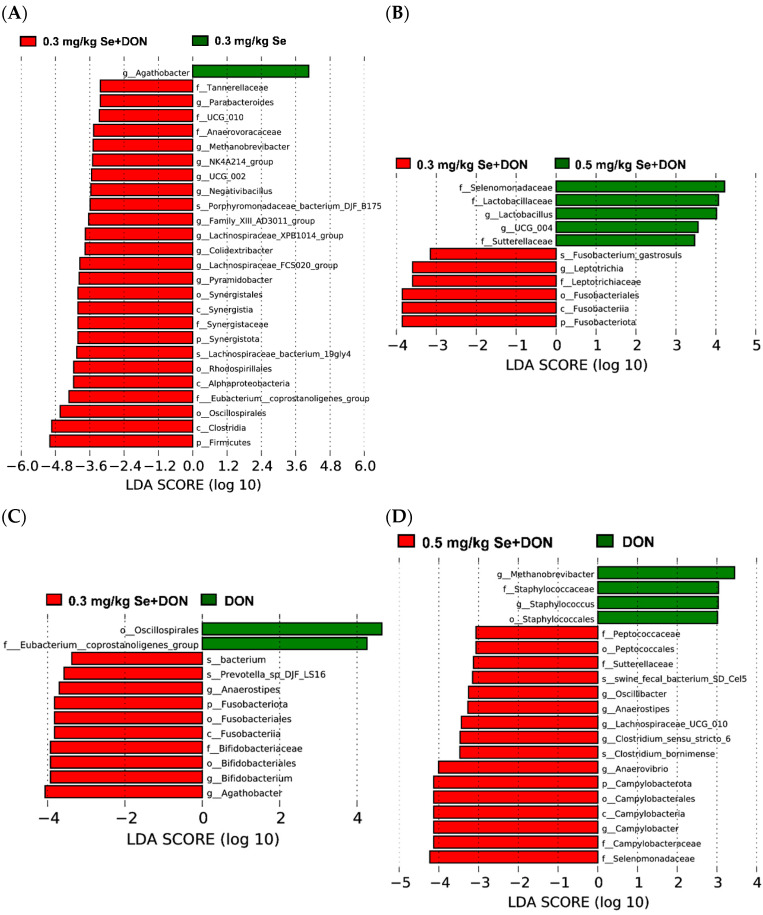
Linear discriminant analysis (LDA) effect size analysis of cecum microbial community alterations (*n* = 6) (LDA scores > 3.0). (**A**) The 0.3 mg/kg Se + DON group vs. 0.3 mg/kg Se group. (**B**) The 0.3 mg/kg Se + DON group vs. 0.5 mg/kg Se + DON group. (**C**) The 0.3 mg/kg Se + DON group vs. 0 mg/kg Se + DON group. (**D**) The 0.5 mg/kg Se + DON group vs. 0 mg/kg Se + DON group.

The LEfSe analysis was further conducted to identify specific microbiota alterations at the phylum (p), class (c), order (o), family (f), genus (g), and species (s) levels (Figure 5). Compared to the 0.3 mg/kg Se group, the 0.3 mg/kg Se + DON group showed a decreased abundance of *Agathobacter* (g), while the abundance of Tannerellaceae (f), *Parabacteroides* (g), UCG_010 (f), Anaerovoracaceae (f), *Methanobrevibacter* (g), *NK4A214_group* (g), *UCG_002* (g), *Negativibacillus* (g), *Porphyromonadaceae_bacterium_DJF_B175* (s), *Family_Xlll_AD3011_group* (g), *Lachnospiraceae_XPB1014_group* (g), *Colidextribacter* (g), *Lachnospiraceae_FCS020_group* (g), *Pyramidobacter* (g), Synergistales (o), Synergistia (c), Synergistaceae (f), Synergistota (p), *Lachnospiraceae_bacterium_19gly4* (s), Rhodospirillales (o), Alphaproteobacteria (c), Eubacterium_coprostanoligenes_group (f), Oscillospirales (o), Clostridia (c), and Firmicutes (p) was elevated (Figure 5A). Compared with the 0.3 mg/kg Se + DON group, the 0.5 mg/kg Se + DON group exhibited decreased abundance of *Fusobacterium_gastrosuis* (s), *Leptotrichia* (g), Leptotrichiaceae (f), Fusobacteriales (o), Fusobacteriia (c), and Fusobacteriota (p). In contrast, the abundance of Selenomonadaceae (f), Lactobacillaceae (f), *Lactobacillus* (g), *UCG_004* (g), and Sutterellaceae (f) was increased (Figure 5B). Compared to the 0 mg/kg Se + DON group, the 0.3 mg/kg Se + DON group had decreased abundance of Oscillospirales (o) and Eubacterium_coprostanoligenes_group (f), but increased abundance of *bacterium* (s), *Prevotella_sp_DJF_LS16* (s), *Anaerostipes* (g), Fusobacteriota (p), Fusobacteriales (o), Fusobacteriia (c), Bifidobacteriaceae (f), Bifidobacteriales (o), *Bifidobacterium* (g), and *Agathobacter* (g) (Figure 5C). Compared with the 0 mg/kg Se + DON group, the 0.5 mg/kg Se + DON group exhibited decreased abundance of *Methanobrevibacter* (g), Staphylococcaceae (f), *Staphylococcus* (g), and Staphylococcales (o), while the abundance of Peptococcaceae (f), Peptococcales (o), Sutterellaceae (f), *swine_fecal_bacterium_SD_Cel5* (s), *Oscillibacter* (g), *Anaerostipes* (g), *Lachnospiraceae_UCG_010* (g), *Clostridium_sensu_stricto_6* (g), *Clostridium_bornimense* (s), *Anaerovibrio* (g), Campylobacterota (p), Campylobacterales (o), Campylobacteria (c), *Campylobacter* (g), Campylobacteraceae (f), and Selenomonadaceae (f) was increased (Figure 5D).

### 3.4. Predicted Metabolic Functions of Cecum Microbiota

**Figure 6:**
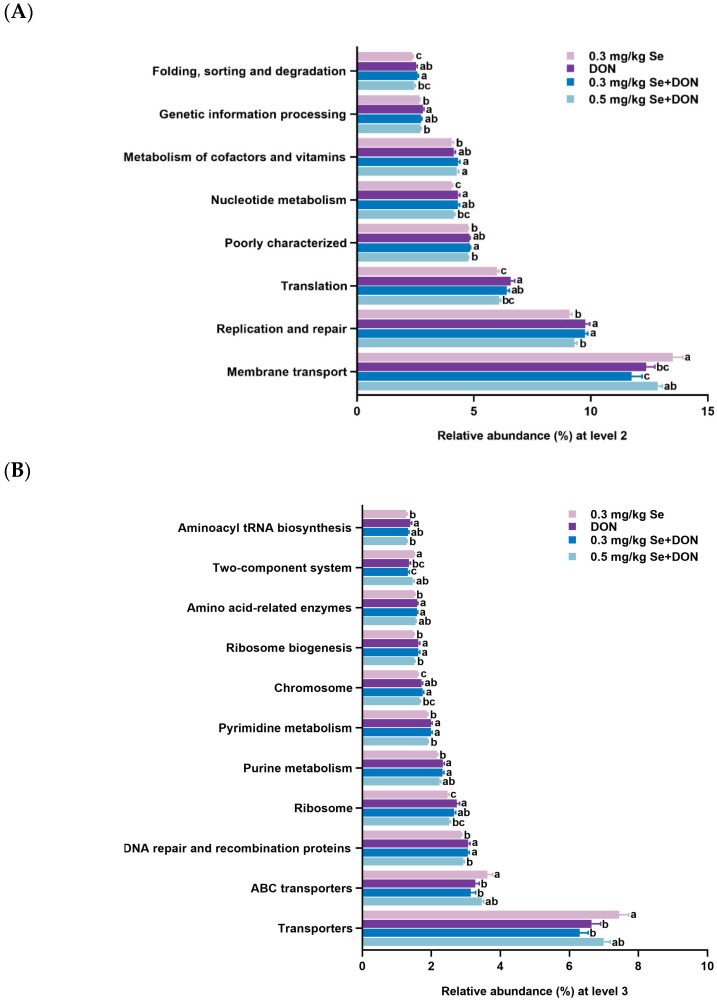
The predicted metabolic functions for the altered genes of cecum microbial communities (KEGG pathways) at metabolism level 2 (**A**) and metabolism level 3 (**B**) (*n* = 6, mean ± SEM). Different letters above the bars indicate statistical differences among the treatment groups (*p* < 0.05).

The PICRUSt analysis was conducted to predict the metabolic functions of the cecum microbiota in the nursery piglets in response to the four dietary treatments (Figure 6). At metabolism level 2, piglets in the 0.3 mg/kg Se + DON group exhibited decreased membrane transport and increased replication and repair, translation, poorly characterized functions, nucleotide metabolism, metabolism of cofactors and vitamins, and folding, sorting, and degradation compared to the 0.3 mg/kg Se group (*p* < 0.05). Conversely, piglets in the 0.5 mg/kg Se + DON group showed increased membrane transport and decreased replication and repair, poorly characterized functions, and folding, sorting, and degradation compared to the 0.3 mg/kg Se + DON group (*p* < 0.05). However, no significant differences were observed in these predicted metabolic functions between the 0.3 mg/kg Se + DON and 0 mg/kg Se + DON groups (*p* > 0.05).

At metabolism level 3, piglets in the 0.3 mg/kg Se + DON group had decreased transporters, ABC transporters, and two-component systems, while showing increased DNA repair and recombination proteins, ribosome, purine metabolism, pyrimidine metabolism, chromosome, ribosome biogenesis, and amino acid-related enzymes compared to the 0.3 mg/kg Se group (*p* < 0.05). In contrast, piglets in the 0.5 mg/kg Se + DON group exhibited increased two-component systems and decreased DNA repair and recombination proteins, pyrimidine metabolism, chromosome, and ribosome biogenesis compared to the 0.3 mg/kg Se + DON group (*p* < 0.05). However, no statistical differences were found in these predicted metabolic functions between the 0.3 mg/kg Se + DON and 0 mg/kg Se + DON groups (*p* > 0.05).

### 3.5. Correlation Analysis of Cecum Microbiota and ATTD of Nutrients

**Figure 7:**
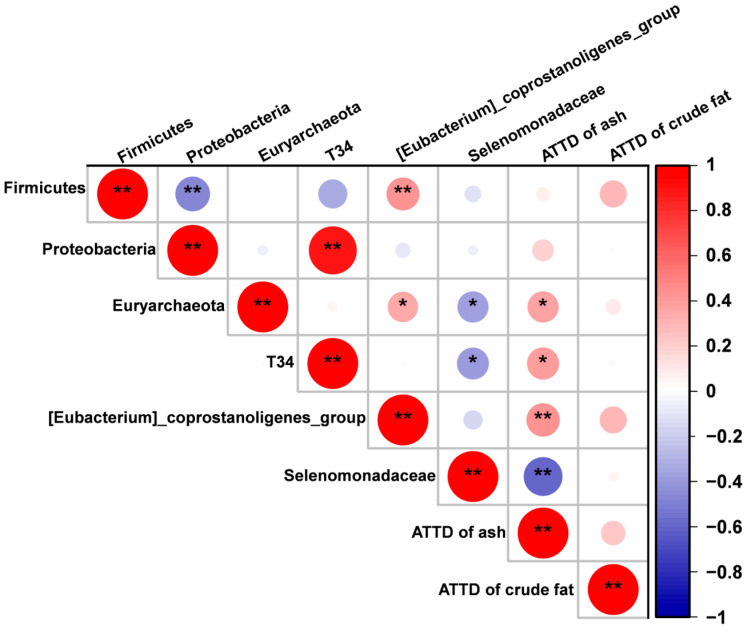
Correlation analysis of the altered cecum microbiota and ATTD of nursery piglets using Pearson correlation analysis. * *p* < 0.10, ** *p* < 0.05.

Pearson correlation analysis was conducted to examine the correlation relationships between the cecum microbiota and ATTD of nutrients (Figure 7). The results indicated that the abundance of Selenomonadaceae was negatively correlated with the ATTD of ash, whereas the abundance of [Eubacterium]_coprostanoligenes_group was positively correlated with the ATTD of ash (*p* < 0.05). Additionally, the abundance of T34 and Euryarchaeota showed a tendency toward positive correlation with the ATTD of ash (*p* < 0.10).

## 4. Discussion

The objective of this study was to evaluate the effects of dietary Se supplementation levels on the ATTD of nutrients and cecum microbiota in nursery piglets challenged with commercial purified DON. To ensure consistency and minimize variability in feed intake, the piglets were fed experimental diets with standardized daily feed allotments of 320, 400, 500, and 600 g over periods of 1, 2, 3, and 4 weeks, respectively. Consequently, the average daily feed intake and average daily gain of piglets did not differ among treatment groups. DON contamination has been reported to reduce nutrient digestibility in pigs. For instance, it was observed that the ATTD of dry matter, gross energy, and crude protein in nursery piglets was decreased when fed diets containing 5.15 mg/kg DON compared to 1.45 mg/kg DON [33]. In this study, we found that, compared with the 0.3 mg/kg Se group, piglets in the 0.3 mg/kg Se + DON (3.0 mg/kg) group exhibited a reduced ATTD of crude ash, while those in the 0 mg/kg Se + DON group showed a decreased ATTD of crude fat. The findings suggest that DON exposure reduced nutrient digestibility in nursery piglets, and this effect was exacerbated by Se deficiency, further impairing nutrient digestibility in pigs. The results further indicated that piglets in the 0.5 mg/kg Se + DON group exhibited a higher ATTD of crude fat compared to the 0 mg/kg Se + DON group, as well as a greater ATTD of crude ash relative to the 0.3 mg/kg Se + DON group. Therefore, under DON exposure conditions, Se sufficiency (without Se supplementation) reduced nutrient digestibility, whereas elevated dietary Se levels enhanced the nutrient digestibility of crude fat and crude ash in nursery pigs.

The cecum serves as the primary location for microbiota digestibility in swine [10]. Therefore, the cecal digesta was selected for the microbial diversity and composition analysis. The effects of DON on gut microbiota may arise from either the direct antimicrobial properties of this mycotoxin or as a secondary consequence of its toxic impact on intestinal cells, leading to the subsequent release of antimicrobial compounds [34]. In the present study, the α-diversity was unaffected in the 0.3 mg/kg Se + DON group compared to the 0.3 mg/kg Se group. However, compared with the 0.3 mg/kg Se group, the Shannon and Chao 1 indices were reduced in the cecal microbiota of nursery piglets from the 0 mg/kg Se + DON group. These findings suggest that under DON exposure conditions, Se deficiency (without Se supplementation) decreased cecal microbiota diversity. Interestingly, elevated Se at 0.5 mg/kg increased the diversity of cecal microbiota, as demonstrated by higher Shannon and Chao 1 indices in pigs from the 0.5 mg/kg Se + DON group compared to the 0 mg/kg Se + DON group. This highlights the role of dietary Se in regulating the diversity of gut microbiota in animals [35].

At the phylum level, compared to the 0.3 mg/kg Se group, piglets in the 0.3 mg/kg Se + DON group showed a reduced relative abundance of Firmicutes and an elevated relative abundance of Proteobacteria in the cecum. These findings align with those of Zhai et al. (2022), who observed that intragastric administration of 10 mg DON/kg body weight for 42 days decreased Firmicutes abundance and increased Proteobacteria abundance in the feces of laying hens [36]. Additionally, in our study, piglets in the 0.5 mg/kg Se + DON group exhibited a higher relative abundance of Firmicutes compared to the 0.3 mg/kg Se + DON group, along with a lower relative abundance of Euryarchaeota relative to the 0 mg/kg Se + DON group. These findings align with those of Li et al. (2021), who reported that dietary supplementation with organic Se (2-hydroxy-4-methylselenobutanoic acid) increased Firmicutes abundance in the colonic microbiota of gilts [27]. These findings were further confirmed by LEfSe analysis. At the family level, the relative abundance of Selenomonadaceae was higher in piglets from the 0.5 mg/kg Se + DON group compared to the other three treatment groups. Supporting our results, Sun et al. (2023) reported that administration of 0.15 mg Se as selenized glucose per liter via drinking water increased the relative abundance of Selenomonadaceae at the family level in the feces of Sprague-Dawley rats [37].

The PICRUSt analysis was conducted to infer the metabolic potential of cecal microbiota in nursery piglets subjected to four dietary treatments. At level 2 of metabolic pathway classification, piglets in the 0.3 mg/kg Se + DON group demonstrated reduced membrane transport alongside elevated functions in replication and repair, translation, poorly characterized processes, nucleotide metabolism, cofactor and vitamin metabolism, and folding, sorting, and degradation relative to those receiving 0.3 mg/kg Se alone. These findings further indicate that DON exposure disrupts gut microbiota, thereby negatively influencing their metabolic functions [38]. However, an elevated Se level of 0.5 mg/kg alleviated the metabolic dysfunctions induced by DON exposure, as evidenced by enhanced membrane transport activity but reduced functions related to replication and repair, poorly characterized processes, and folding, sorting, and degradation compared to the 0.3 mg/kg Se + DON group. Consistently, at metabolism level 3, piglets in the 0.3 mg/kg Se + DON group exhibited reduced levels of transporters, ABC transporters, and two-component systems, while displaying elevated levels of DNA repair and recombination proteins, ribosome activity, purine metabolism, pyrimidine metabolism, chromosome function, ribosome biogenesis, and amino acid-related enzymes compared to the 0.3 mg/kg Se group. Conversely, piglets in the 0.5 mg/kg Se + DON group demonstrated increased two-component systems and decreased DNA repair and recombination proteins, pyrimidine metabolism, chromosome function, and ribosome biogenesis relative to the 0.3 mg/kg Se + DON group. The results further indicate the role of Se in regulating gut microbiota and their metabolic functions [39].

Pearson correlation analysis was conducted to evaluate the correlation between cecal microbiota composition and the ATTD of the measured nutrients. The findings revealed a significant negative correlation between Selenomonadaceae abundance and the ATTD of ash, while a positive correlation was observed between [Eubacterium]_coprostanoligenes_group abundance and the ATTD of ash. As reported by Reyer et al. (2021), the colonic Selenomonadaceae abundance negatively correlates with the serum phosphorus levels in piglets [40]. [Eubacterium]_coprostanoligenes_group was also reported as one of the primary genera of gut microbiota influencing nutrient digestibility in pigs [41]. This further supports our results that Se mitigates the adverse effects of DON exposure on the nutrient digestibility of piglets, which is partially associated with cecum microbiota.

Lastly, aside from cecal microbiota digestibility by Se, the increased ATTD of crude ash and crude fat in piglets (monogastric animals) due to elevated Se supplementation may be attributed to potential biochemical and physiological pathways, including Se’s role in antioxidant defense systems and the enhancement in digestive enzyme activity. Indeed, our previous studies demonstrated the antioxidant defense function of Se in gestating and lactating sows [12,13], as well as in late-gestating and lactating sows [42], for offspring piglets, and in finishing pigs [43]. Elevated antioxidant defense may assist in promoting nutrient digestibility in pigs [44]. These findings were supported by the studies by Qin et al. (2023) [45] and Wang et al. (2023) [46], which demonstrated that dietary Se supplementation enhanced the digestive enzyme activities in piglets. Therefore, the elevated dietary Se level enhanced the nutrient digestibility of nursery piglets exposed to DON, which is partially attributed to the modulation of cecal microbiota diversity and composition.

## 5. Conclusions

In conclusion, an elevated dietary Se level of 0.5 mg/kg enhanced the nutrient digestibility of crude ash of nursery piglets exposed to DON, an effect apparently linked to the modulation of cecal microbiota diversity and composition.

## Figures and Tables

**Table 1:** Dietary treatment groups.

Items	Dietary Treatment Groups			
	Group 1	Group 2	Group 3	Group 4
DON supplementation level ^1^	0 mg/kg	3.0 mg/kg	3.0 mg/kg	3.0 mg/kg
Se supplementation level ^2^	0.3 mg/kg	0 mg/kg	0.3 mg/kg	0.5 mg/kg

^1^ Deoxynivalenol (DON) was supplemented as purified DON (purity ≥ 98%). ^2^ The selenium (Se) supplementation levels were achieved by adjusting the mineral premix using individual minerals, including selenomethionine, ZnSO_4_·H_2_O, FeSO_4_·H_2_O, MnSO_4_·H_2_O, CuSO_4_·5H_2_O, CaI_2_O_6_, and zeolite as a carrier. Purified selenomethionine (purity ≥ 99%) was used to prepare diets supplemented with 0.3, 0, 0.3, and 0.5 mg/kg Se. It should be noted that the Se levels in the four dietary treatments were 0.3, 0, 0.3, and 0.5 mg/kg, excluding the background Se content (0.03–0.06 mg/kg Se) present in the feed ingredients.

## Data Availability

The raw sequencing data for cecal microbiota were archived in the National Center for Biotechnology Information (NCBI) under the accession number PRJNA1307152.
